# Tuberculosis treatment delay and associated factors among pulmonary tuberculosis patients at public health facilities in Dale District and Yirgalem Town administration, Sidama Region, South Ethiopia

**DOI:** 10.1186/s12879-024-09397-8

**Published:** 2024-05-23

**Authors:** Daniel Dawit Rima, Derese Legese, Endrias Markos Woldesemayat

**Affiliations:** 1 Sidama Region Health Department, Sidama Region, Hawassa, Ethiopia; 2 School of Public Health, College of Medicine and Health Science, Hawassa University, Hawassa, Ethiopia

**Keywords:** Tuberculosis, Treatment delay, Dale, Yirgalem, Sidama, South Ethiopia

## Abstract

**Background:**

Tuberculosis (TB) treatment delay is one of the major challenges of TB care in many low-income countries. Such cases may contribute to an increased TB transmission and severity of illness. The aim of this study was to determine the magnitude of patient delay in TB treatment, and associated factors in Dale District and Yirgalem Town administration of Sidama Region, Southern Ethiopia.

**Methods:**

Between January 1-Augst 30/ 2022, we studied randomly selected 393 pulmonary TB cases on Directly Observed Treatment Short course (DOTS) in Dale District and Yirgalem Town Administration. After conducting a pretest, we interviewed participants on sociodemographic, health seeking behavior and clinical factors and reviewed the TB registry. Trained enumerators interviewed to collect data. We entered data in to EPI-info 7 version 3.5.4 and then exported to the Statistical Package for Social Science (SPSS) version 23 for analysis. Multivariable logistic regression was used to identify associated factors of TB and statistical significance was defined using the 95% confidence interval.

**Result:**

A total of 393 (98%) participants involved in the study. The magnitude of delay in TB treatment among the study participants was 223 (56.7%) (95% CI (51.8 – 61.6%)). Distance of the health facility from home, (adjusted odds ratio (AOR) = 2.04, 95% CI (1.3, 3.2)), seeking antibiotic treatment before being diagnosed for TB (AOR = 2.1, 95% CI (1.3, 3.5)) and the knowledge of TB prevention and treatments (AOR = 5.9, 95% CI (3.6, 9.8)), were factors associated with delay in TB treatment.

**Conclusion:**

The prevalence of TB treatment delay among pulmonary TB patients in the study setting was high. Delay in TB treatment was associated with knowledge, behavioral and accessibility related factors. Providing health education and active case finding of TB would help in minimizing the delay.

**Supplementary Information:**

The online version contains supplementary material available at 10.1186/s12879-024-09397-8.

## Background

According to the Global tuberculosis (TB) report 2022, an estimated 9.9 million people were diseased with TB in 2020 [1]. Ethiopia is one of the 30 high TB burden countries. A recent report from the country showed that more than half of TB patients faced having delay in treatment-seeking [2]. Delay in seeking treatment among TB patients is one of the major challenges of TB care in most low-income countries like Ethiopia [3]. Such cases may be contributing to an increased TB transmission and severity of illness.

Delay in TB treatment could be due to patient delay which consists of the time elapsed between onset of TB symptoms and first self-presentation to formal health care or provider delay which comprises of the time elapsed between first presentation to formal care and anti-TB treatment initiation. Identifying the magnitude and factors associated with patient delay in treatment-seeking will help to improve TB control by improving case findings, providing early treatment, and reducing infection sources in the community [2, 4].

The magnitude of patient delay in TB treatment was 69% in South India in 2014 [5], 72% with greater than 30 days delay in Colombia in 2014 [6], 34.8% with a median delay of 32 days in Turkey in 2019 [7], 65.5% in Iran in 2018 [8], and 61 and 30 days in Peruvian Amazon in 2012 and Thailand in 2008 [9, 10], respectively. African studies reported prevalence of 61% in Nigeria in 2012 [11] and 48% in Zimbabwe in 2014 [12]. A cross-sectional study in Bamako, Mali reported the median patient delay of TB treatment was 58 days [13]. Over half (56.3%) of all involved patients delay in seeking health care for more than 30 days after the onset of their TB symptoms [14]. In a cross-sectional study most (57.8%) of the TB diagnosis delayed for over 21 day [15].

Reports from Ethiopia showed that TB treatment delay was 62.3% in the North Wollo Zone in 2015 [16], 42% in Addis Ababa in 2016 [17], 50.9% in Gedeo Zone in 2020 and 56.9% Gamo Zone in 2017 [18, 19]. The pooled prevalence of TB treatment seeking delay was 44.29% in Ethiopia [20]. The median patient delay in TB diagnosis in Hadiya Zone, South Ethiopia was 30 days [21]. A meta-analysis paper showed that the median time of patient delay was 24.6 days [22]. A recent report also confirmed that the median patient delay was higher among pastoralists (35.5 days) than among non-pastoralists [23].

Concerning factors associated with patient delay in TB treatment, low income, rural residency, unemployment, old age, and female sex, distance to the DOTs centers, low education level, social reasons and non-request of health workers were the associated factors of patient delay in TB treatment [13, 24, 25]. Factors like lack of knowledge, fear and embarrassment of receiving TB diagnosis, patient tendency to get self-treatment before seeking formal medical care, use of dispensary and private health facilities, employed individuals, secondary-level education and tertiary education, body mass index (BMI) status were also reported as risk factors of patient related TB treatment delay in other settings [15, 18, 26]. The overall TB knowledge was not statistically associated with seeking health care for TB diagnosis [14], while other treatment seeking before formal health care provider, residence of the patient, the type of TB, educational level, distance from the DOTS center, lack of awareness about TB, consulting non-formal health provider, and being rural resident were associated factors of treatment delay reported by other studies [20, 22]. Socioeconomic and perception related factors, urban residence, religious views, low income, misconception about the time of TB treatment to be cured and lack of comfort with DOTS were associated with patient delay in TB diagnosis [21].

There is a recent report that estimated the magnitude of TB treatment delay among TB patients on DOTS in Hawassa City Health Centers in Sidama Region [27]. These cases consisted of TB cases mainly coming from urban areas. However, there is no report from the current study area, Yirgalem town and Dale District in the Sidama Region, which consists of both urban and rural population, and where the incidence and prevalence of TB are high. Bacteriological confirmed TB case notification rate in Sidama Region was 163 per 10^5^populations in 2022 [28]. Dale district and Yirgalem town are among the districts with the highest burden. The aim of this study was to determine the magnitude of patient delay in TB treatment, and associated factors in Dale District and Yirgalem Town administration of Sidama Region, Southern Ethiopia.

## Methods and materials

### Study design, setting and period

The study design used in the current study was a facility-based cross-sectional study conducted during the period from January 1 to Augst 30/2022 in Dale District and Yirgalem Town Administration of the Sidama region, South Ethiopia. Sidama Region constitutes over four million people. The administrative town of Dale district; Yirgalem is located at about 47 km away from Hawassa, the Region capital and at 321 km to the south of Addis Ababa, the capital city of Ethiopia. Dale district consists mainly rural population which has a total population of 254,652 residing in 34 kebeles (smallest administrative unit). Regarding the health care facility, the district has one General Hospital, twelve health centers, 34 health posts, and 2 private clinics, and 3 drug vendors. At the time we collected data, all government health facilities in the district are providing DOTs by trained health workers. All pulmonary TB patients on DOTs during the study period, and registered at public health facilities in the districts were considered as our source population. Randomly selected pulmonary adult TB cases at least fifteen years of age were considered as the study population. Pulmonary TB cases who were critically ill, and not volunteer to be interviewed due to their illness were excluded from the study.

### Sample size and sampling procedures

**Fig. 1:**
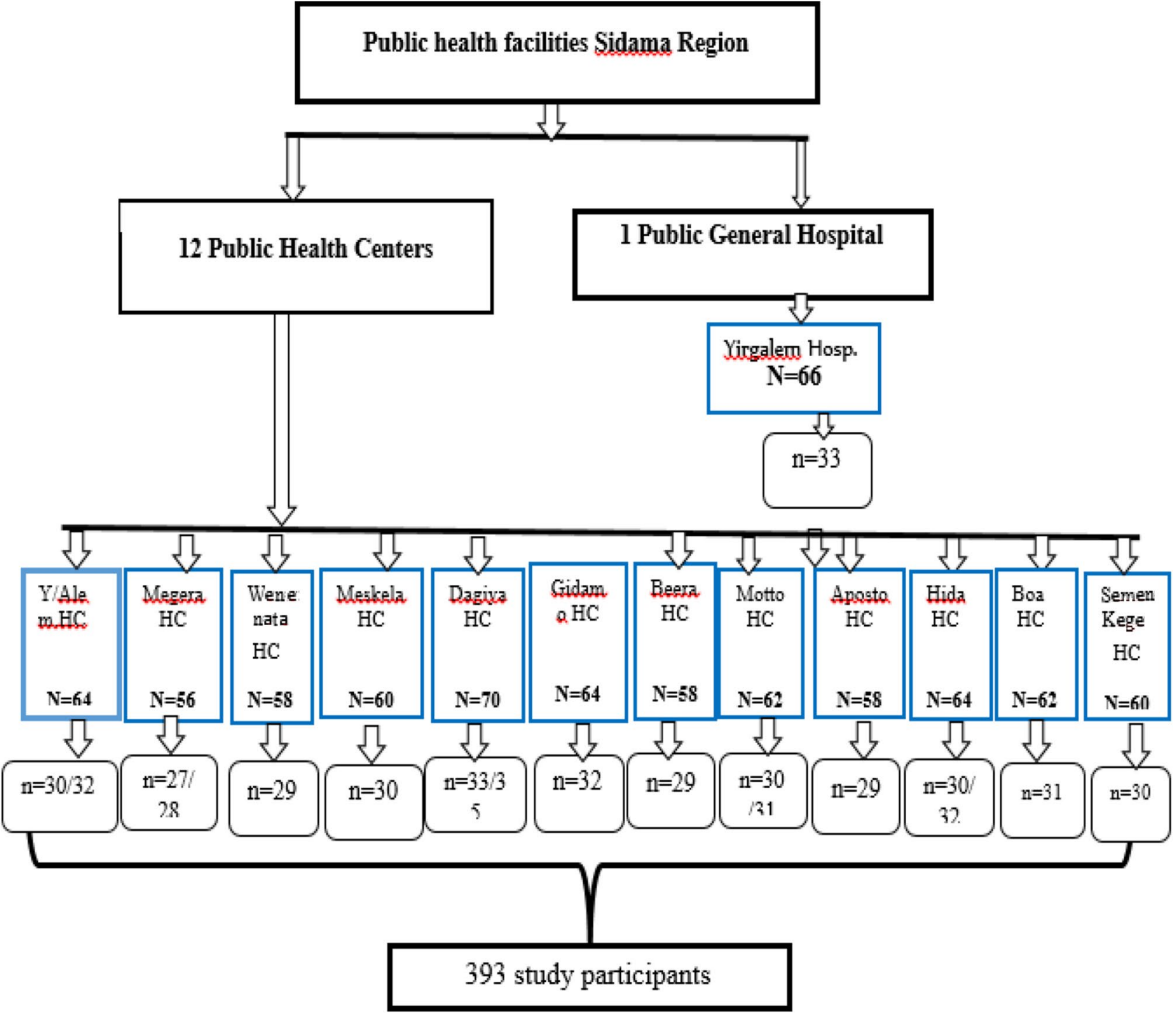
Schematic presentation of the sampling procedure *NB: HC = health center, N = total number of pulmonary tuberculosis getting treatment from the facility, n = total number of sampled tuberculosis cases from the facilities, Hosp. = Hospital*

Sample size was calculated by using single population proportion formula. A proportion of 50.9% delay in TB treatment in Gedeo Zone, Southern Ethiopia was considered [19]. Using a 95% confidence interval (CI), 5% of marginal error and 5% non-response rate, the minimum sample size estimated for the study was 403 TB cases. We included Yirgalem General Hospital and all of the 12 health centers to recruit the study participants. Proportional allocation of TB cases was done according to the number of TB cases in each of the health facilities. Figure 1 shows schematic presentation of the sampling procedure. Patients who were on follow up during the data collection period were listed by using the patients’ medical registration number and then participants were selected using simple random sampling method from the list. Then when patients came to the facilities to receive their TB care they were interviewed. Data were collected until the required sample size was achieved.

### Variables of the study

The dependent variables considered in the study was patient delay in TB treatment. Independent variables were socio-demographic characteristics such as age, sex, ethnicity, marital status, religion, occupation, residence area, educational level, family size and family monthly income. Health seeking behavior of patients, clinical characteristics and knowledge on TB treatment were other independent variables considered in the study.

### Data collection tools and procedure

We mainly used a pre-tested and interviewer-administered questionnaire to collect the data. Data on smear positivity and X-rays were obtained by record review. The instrument was developed after reviewing of previous studies [16–19]. We asked participants socio-demographic characteristics related variables. Questions about clinical factors, visiting other health facilities and knowledge on TB treatments were also included in the questionnaire. Supplementary material (S1) shows the tool we used for knowledge related factors assessment.

The questionnaire was first prepared in English and then it was translated in to the local language (Sidamu afoo) and then it was back translated in to English to check the consistencies in meaning. The Sidamu afoo version questionnaire was used for the data collection purpose. Thirteen health professionals with a B.Sc. degree in nursing or public health working in the facilities and 2 supervisors with MPH training working in the district offices, were assigned to collect the data. The data collectors were fluent speakers of both Amharic and Sidamu Afo.

### Data quality control

Data collectors and supervisors were trained on issues related to the research aim, data collection methods, and data collection tool. The collected data were reviewed on daily basis. Data completeness and missing value were checked before analysis. The questionnaire was pretested on 5% of sample size in the neighboring Wonsho District, which was not included in the study. Based on the pre-test finding, logical sequence, clarity of the questionnaire and the questions that had made confusion for the respondents were revised. An updated questionnaire was used to collect the final data. During the data collection process, any problem encountered was discussed with the team and were solved immediately.

### Operational definitions

#### Pulmonary TB cases

Refers to patients with two or more sputum smears or gene x-pert positive for acid-fast bacilli were considered as smear-positive pulmonary TB cases. Cases diagnosis by clinical and x-ray evidences (smear negative by Zehil Nelson Staining or gene x-pert) were considered as smear-negative pulmonary TB cases [29].

#### Patient delay

Refers to the time interval between onset of symptoms mainly having of cough for over 2–3 weeks and the patient’s first contact with a healthcare provider. If TB cases are presented to modern health facility thirty days after initial start of the TB symptom considered as delayed [30, 31].

#### Knowledge

Patients who scored more than the set average (50%) were considered knowledgeable and those who scored less than average were considered as low knowledgeable [19].Variables measuring knowledge were recorded on a 3-point Likert scale of 10 questions. These include by asking questions on the knowledge about the type of disease, its causes, curability, and type of anti TB drugs, and the duration of treatment. Knowledge related factors assessment tool is submitted as a supplementary material (supplementary material 1).

### Data analysis procedure

Data were coded and entered in to EPI-info 7 version 3.5.4 statistical software and then exported to the Statistical Package for Social Science (SPSS) version 23. Data were cleaned. The data were presented by using tables. Bivariable and multivariable logistic regression analysis were used to show relationship between variables. Independent variables with a p-value less than or equal to 0.25 in the bi-variable were entered in to multivariable logistic regression model to control the effects of confounders. The presence of association between predictor variables and delay in TB treatment was assessed using a 95% confidence interval and p-value.

## Results

### Socio-demographic characteristics of participants

A total of 393 pulmonary TB patients were included in this study with a 97.5% response rate. Eight patients were excluded from the study, since they were seriously ill and or not volunteer to respond. Most of the participants, 217 (55%) were males, and 157 (40%) were in the age group of 15–35 years. The mean (standard deviation (SD)) age of the study participants was 29.92 (± 9.65) years. Two hundred and forty four (62%) of the participants were residing in rural areas, 266 (67.7%) were married, 146 (37%) of them were not attending formal education (see Table 1).

**Table 1:** Socio-demographic characteristics of pulmonary tuberculosis patients

Characteristics	Category	Number	Percent
Age in years	15–25	157	40
	26–35	144	37
	36–45	53	13
	>45	39	10
Sex	Male	217	55
	Female	176	45
Marital status	Married	266	67.7
	Single life*	127	32.3
Educational status	No attended formal education	146	37
	Primary school	96	24
	Secondary school	93	24
	College or University	58	15
Ethnicity	Sidama	284	72
	Amhara	27	7
	Oromo	24	6
	Others	58	15
Religion	Protestant	300	76
	Orthodox	66	17
	Muslims	27	7
Place of Residence	Rural	244	62
	Urban	149	38
Occupation	Farmer	151	38.5
	Employed	49	12.5
	Daily labor	40	10
	Student	129	33
	Merchant	24	6
Household monthly income	< 1000 ETB	188	48
	*≥* 1000 ETB	205	52
Family size in house holds	< 5 people	226	57.5
	*≥* 5 people	167	42.5

* Single life: single, widowed and divorced, ETB: Ethiopian Birr (exchange rate 1 USD = 48 ETB)

### Knowledge & behavior related characteristics

In this study, 8 (1.9%) of the study participants were current cigarette smoker and 30 (8%) were currently drinking alcohol, and 209 (53%) of study participants had low knowledge of TB. Table 2 shows the knowledge and behavior related characteristics of the study participants.

**Table 2:** Tuberculosis treatment delay, knowledge score and behavioral characteristics of the study participants

Variables	Category	Number	Percent
Smoking status	Never smoke	386	98.2
	Current smoker	7	1.8
Alcoholic drinking status	Never drink	363	92
	**Currently drinking**	30	8
Tuberculosis knowledge score	Good	184	47
	Poor	209	53
Tuberculosis treatment delay	No delay	170	43.3
	Delay	223	56.7

### Clinical characteristics, health care accessibility and health seeking behaviors of the patients

About one-third, 120 (30.5%) of the study participants had previous history of TB. Regarding patient condition before first visit to the facilities, 231 (58.8%) cases were actively working. Among the studied people, 298 (76%) were smear-positive cases and 93 (24%) cases were diagnosed by clinical evaluation and X-ray. The primary symptoms that patients experienced during the onset of their illness was coughing, 272 (69.2%). The magnitude of delay in TB treatment among the study participants was 223 (56.7%) (95% CI (51.8 – 61.6%), with the median (interquartile range) of delay in TB treatment of 30 (20–45) days (Table 3).

**Table 3:** Clinical characteristics and health seeking behaviors of tuberculosis patients

Variables	Category	Number	%
History of TB treatment	Yes	120	30.5
	No	273	69.5
Patient condition before first visit to facilities	Actively working	231	58.8
	Some activity outside house	117	29.8
	Limited at house/bed ridden	45	11.5
Diagnosis of TB	Sputum examination	298	75.8
	X-rays	95	24.2
Sign and symptom of infected with TB	Cough of > 2weeks	272	69.2
	Weight loss	48	12
	Loss of appetite	42	10.7
	Fever	31	7.8
Consultation before visit to facilities for the current diseases	Public health facility	339	86
	Formal private health facility	38	10
	Informal private health facility	16	4
Patients given drugs other than anti-TB drugs at 1st visit	Yes	284	72
	No	109	28
Distance from the nearest health facility	Mean (SD)	5.8 (3.4)	
Home distance from the nearest health facility	< 5 Kms	138	44
	*≥* Kms	216	56
Presence of other health facilities close to home	Yes	231	58.8
	No	117	29.8
Number of health providers visited for current illness	≤ 2 health care providers	298	76
	≥ 3 health care providers	93	24
Anti-biotic treatments before TB diagnosis	Yes	272	69.2
	No	48	12

*Kms = kilometers, TB = tuberculosis, SD = standard deviation*

Of all the study participants, 216 (56%) TB cases travel over an estimated 5 km to reach to the nearest health facility from their home, with the mean (SD) of 5.8 (3,4) kilometers. High proportion, 272 (69.2%) of the study participants were treated with antibiotic before being diagnosis with TB in the health facilities (Table 3).

### Factors associated with patient delay for TB treatment

In a bivariable logistic regression analysis place of residence, distance of the health facility, seeking antibiotic treatment before being diagnosed for TB, and knowledge of TB prevention and treatment, have shown association with patient delay in TB treatment. In a multivariable logistic regression analysis however, distance of the health facility from home, (adjusted odds ratio (AOR) = 2.0, 95% CI (1.3, 3.2)), seeking antibiotic treatment before being diagnosed for TB (AOR = 2.1, 95% CI (1.3, 3.5)), and the knowledge of TB prevention and treatments (AOR = 5.9, 95% CI (3.6, 9.8)), were significantly associated with delay in TB treatment (Table 4).

**Table 4:** Factors affecting patient delay in tuberculosis treatment in Dale district

Variables	Treatment delay			
	Yes	No	COR (95% CI)	AOR (95% CI)
Sex				
Male	114	103	0.6 (0.4,1.0)	0.7 (0.4,1.1)
Female	109	67	1	1
Marital status				
Married	143	123	0.7 (0.4, 1.1)	0.6 (0.4 1.1)
Single life	80	47	1	1
Education				
Informal education	81	65	0.5 (0.2,1.0)	0.7 (0.3,1.4)
Primary school	52	44	0.5 (0.2, 1.0)	0.6 (0.3, 1.2)
Secondary school	50	43	0.5 (0.2, 1.0)	0.7 (0.3, 1.6)
Higher school	40	18	1	1
Residence				
Rural	127	117	0.6 (0.3, 0.9)	0.6 (0.4, 1.0)
Urban	96	53	1	1
Distance from home				
< 5 Km	87	84	1	1
≥ 5 Km	136	86	1.5 (1.2, 2.2)	1.9 (1.2, 3.1)
Antibiotic treatment before diagnosis				
Yes	278	167	1.6 (1.0, 2.5)	2.1 (1.3,3.4)
No	56	59	1	1
Tuberculosis knowledge score				
Poor	66	118	5.3 (3.4, 8.3)	6.2 (3.9, 10.0)
Good	157	52		

*AOR: Adjusted Odds Ratio, COR: Crude Odds Ratio, CI: Confidence Interval, Km: Kilometer*

## Discussion

Early detection of pulmonary TB cases and treating them are among the strategies of controlling TB. In this study, we found a high median delay time of TB treatment among the study participants. The prevalence of TB treatment delay among pulmonary TB patients in the study setting was high. Delay in TB treatment was associated with being treated with antibiotics before the diagnosis of TB, distance of the health facilities to get the TB care and lack of the knowledge related to TB.

The prevalence of patient delays in treatment of TB in the current study was nearly similar with the study reports from India 55.6.0% [32], Uganda 58% [33], Ghana 60% [34], Gamo Zone, 56.9% (29) and Gedeo Zone in the Southern Ethiopia, 50.9% [19]. However, our result was lower than the study reports from South western Iran 65.5% [6], and North Wollo Zone in North Ethiopia 62.3% [35], but it was higher than the findings from another reports from India, 49% [36] and South Africa, 40% [37]. The possible reasons for these discrepancies is that the difference in culture, socio-economy and time of conducting the studies.

The median patient delay in TB treatment in this study was consistent with the reports from Bahirdar, 30 days [38], Thailand was 30 days [9], Oromia region 35 days [39], and North Wollo Zone 36 days [35]. The present study finding however, is lower than that of the reports from Amara region Gondar town, 41 days [40] and Afar Region, 70 days [41]. This difference may be due to the differences in access to TB care (availability of diagnostic facilities). Another reason might be the difference in population characteristics which could be due to difference in health seeking behavior, the knowledge and geographical area. The current study finding is lower than many African reports. The median patient TB treatment delay was very high in most Africa countries, for instance in Ghana it was 59 days [34], in Uganda 58 days [33], and in Tanzania 48 days [42]. Also, there was a high delay in TB treatment in Peruvian Amazon, 61 days [10].

Concerning factors associated with delays in TB treatment, TB cases residing at a distance of at least 5 km from the nearest health facility were more likely to delay in TB treatment than those who were living below 5 km distance. This result was consistent with reports from Gedeo Zone, Southern Ethiopia [19], South India [5], and Jima Zone, Oromia Region, Ethiopia [32]. TB cases from distant area may face difficulties to access the TB diagnostic and treatment services. They spend more transportation costs than patients from near areas. Thus it is advisable to provide support for patients living at distant place to reach to the health facilities and designing strategies to access such TB cases. One of the strategies could be community based TB case detection [43].

The current study has also showed that TB patients who received antibiotics treatment from somewhere else before the diagnosis of TB were more likely to be delayed in getting TB treatment. This result was consistent with the finding from Afar region [41] and Amhara region [39]. This could be due to the low awareness of the TB cases about TB symptoms. Most of the times, taking antibiotic treatment may initially lower the respiratory symptoms of TB. This could result patients to think that the illness is over and lead to patient to delay to be diagnosed and get timely TB treatment. Therefore, providing health education to such patients may be important to lower the risk of delay in TB treatment.

TB patients with low knowledge of TB had greater risk of delayed TB treatment. The result of this study was consistent with the reports from Thailand, South-India [44], North Wollo, Amhara Region Ethiopia [35], Hadiya Zone, Southern Ethiopia [45], and Oromo Special Zone of the Amhara Region [39]. This finding could be justified by study patients with good knowledge about TB might have a better inclination for early seeking of proper medical care than those without. Having good knowledge of TB mode of transmission, prevention methods, knowledge about the diagnosis & treatment option are vital for early seeking of TB care.

### Strengths and limitations of the study

In this study, we used relatively adequate sample size; as it is shown in Table 4 we have found narrow 95% confidence interval in measuring associations between the dependent and independent variables, especially for distance from home and antibiotic treatment before diagnosis variables. This can be considered as the strength of the study. Using a cross sectional study design which may not confirm the temporal relationship of the cause and effects could be considered as one of the limitations of the study. There may be information bias in the study as the data collection was relied on the participant’s self-reported duration of total delay and therefore this may subject to recall bias. Also there might be social desirability bias, especially on self-reporting of some sensitive issues like cigarette smoking status and alcohol drinking status.

## Conclusions

The study showed that there is high median delay time in TB treatment and a high prevalence of delay in TB treatment among pulmonary TB patients in Dale district and Yirgalem town administration. Living at a distance of over 5 km from the nearest health facility, having low knowledge of TB & being treated with antibiotic before the current TB diagnosis were factors associated with delay in treatment of TB. Improving the health related knowledge of the public by providing health education to the community and solving problems related with service accessibility of health facilities may help to minimize the delay. Active case finding of TB using the health extension workers may also help in minimizing the delay in diagnosis and treatment due to service inaccessibility.

### Electronic supplementary material

Below is the link to the electronic supplementary material.

Supplementary Material 1

## Data Availability

The datasets used and/or analyzed during the current study available from the corresponding author on reasonable request.
