# Supplementary material for: Tuberculosis treatment delay and associated factors among pulmonary tuberculosis patients at public health facilities in Dale District and Yirgalem Town administration, Sidama Region, South Ethiopia
Source: BMC Infect Dis. 2024 May 23;24:517. doi: 10.1186/s12879-024-09397-8 (PMC11112870; doi:10.1186/s12879-024-09397-8)
Supplement: Supplementary file 1 — Supplementary Material 1 [file 12879_2024_9397_MOESM1_ESM.docx]

**S1. Knowledge related factors assessment tool**

| 1 | Do you know what TB is? | 1. Yes 2. No |
| --- | --- | --- |
| 2 | Do you think that TB is a serious disease? | 1. Yes 2. No |
| 3 | What is your own opinion that causes TB? | 1. Infection 2. Punishment  3. Unavoidable 4. Don't know  5. Others (specify)_______ |
| 4 | What are the symptoms of someone infected with TB? | 1. Cough for more than 2 weeks  2. Sputum with blood 3. Fever  4. Weight loss 5. Don't know |
| 5 | Do you believe that TB is caused by mycobacterium TB? | 1. Yes 2. No |
| 6 | Do you think tuberculosis is contagious? | 1. Yes 2. No |
| 7 | Do you believe that TB is transmitted by air? | 1. Yes 2. No |
| 8 | Do you believe that TB is transmitted by hereditary? | 1. Yes 2. No |
| 9 | Do you believe that keeping away from the infected individual can prevent TB transmission? | 1. Yes 2. No |
| 10 | Do you know how TB is diagnosed? | 1. Yes 2. No |
| 11 | If Q No. 610 Yes, how is its diagnosis? | 1. Sputum examination 2. X-ray 3. Other ___ |
| 12 | Do you know that TB is curable? | 1. Yes 2. No |
| 13 | Do you know what some cases of TB will require longer treatment to be cured? | 1. Yes 2. No |
| 14 | Do you know that TB treatment is free? | 1. Yes 2. No |
